# One-year outcome of coenzyme Q10 supplementation in *ADCK3* ataxia (ARCA2)

**DOI:** 10.1186/s40673-019-0109-2

**Published:** 2019-12-16

**Authors:** Tommaso Schirinzi, Martina Favetta, Alberto Romano, Andrea Sancesario, Susanna Summa, Silvia Minosse, Ginevra Zanni, Enrico Castelli, Enrico Bertini, Maurizio Petrarca, Gessica Vasco

**Affiliations:** 1 Department of Neurosciences, Bambino Gesù Hospital, via della Torre di Palidoro, Fiumicino, Rome, Italy; 2 Department of Systems Medicine, University of Roma Tor Vergata, Rome, Italy

**Keywords:** ADCK3, ARCA2, Ataxia, Coenzyme Q10

## Abstract

**Background:**

The recessive ataxia ARCA2 is a rare disorder characterized by Coenzyme Q10 (CoQ10) deficiency due to biallelic mutations in *ADCK3* gene. Despite the pathophysiological role, available data are not univocal on clinical efficacy of CoQ10 supplementation in ARCA2. Here we described the long-term motor outcome of 4 untreated ARCA2 patients prospectively followed-up for one year after starting CoQ10 oral supplementation (15 mg/kg/day).

**Methods:**

Clinical rating scales (SARA; 9 holes peg test; 6 min walking test; Timed 25-Foot Walk) and videoelectronic gait analysis were performed at baseline and every 6 months (T0, T1, T2) to evaluate the motor performances. Since two patients discontinued the treatment at the 7th month, we could provide comparative analysis between longer and shorter supplementation.

**Results:**

At T2, the gait speed (Timed 25-Foot Walk test) significantly differed between patients with long and short treatment; overall, the clinical condition tended to be better in patients continuing CoQ10.

**Conclusions:**

Although preliminarily, this observation suggests that only prolonged and continuous CoQ10 supplementation may induce mild clinical effects on general motor features of ARCA2. Dedicated trials are now necessary to extend and validate such observation.

## Background

Autosomal Recessive Cerebellar Ataxia 2 (ARCA2) is a rare and heterogeneous condition, including cerebellar ataxia, exercise intolerance, epilepsy, dystonia, and mild intellectual disability. Age of onset ranges from typical early childhood to adulthood, while the clinical course is mild or moderate [1, 2]. ARCA2 is due to biallelic mutations of *ADCK3* gene, which encodes for a UbiB family kinase, involved in biosynthesis of Coenzyme Q10 (CoQ10), an electron carrier and endogenous antioxidant. *ADCK3* variants are thus responsible for a primary CoQ10 deficiency, which may play a role in the pathogenesis of the disease; however, several other contributors have been considered, such as the mitochondrial dysfunction or the alteration of neurotransmitters metabolism [3].

The identification of the pathophysiological background prompted several attempts to treat ARCA2 with CoQ10 oral supplementation. The clinical response has been described as inconstant, unpredictable and variable among patients [1, 4], although the genetic background may have a role [3]. Actually, the literature is scarce or anecdotal, and mainly results from retrospective analysis. To date, dedicated trials have not been conducted; as well, dosage and duration of treatments vary among the reports. In addition, the natural history of the disease has not been clearly described yet. Therefore, the unbiased therapeutic efficacy of such intervention can not be established.

In this report, we describe the prospective observation of four untreated ARCA2 patients undergoing standard oral CoQ10 supplementation and systematic evaluation of motor functions by both clinical scores and videoelectronic gait analysis, aiming to outline preliminary but necessary points for the development of dedicated trials (e.g. time of duration, daily dose and outcome measures).

## Methods

Four ARCA2 patients, diagnosed through NGS panel were observed at the Bambino Gesù Children’s Hospital, Rome – Italy (2017–2019). None of them had severe motor disability (Item 1 of SARA, Scale for Assessment and Rating of Ataxia < 4) and intellectual disability (IQ < 55), previous or current CoQ10 supplementation, Brain MRI and nerve conduction study/electromyography abnormalities.

CoQ10 (Ubidecarenone) oral capsule was prescribed, titrated up to 15 mg/kg/die, distributed in three daily administrations at meal time [1] (all patients assumed the same product and continued other medications and physical therapy).

Patients, after the baseline evaluation (T0), started CoQ10 and received two successive visits, every 6 months (T1 and T2 respectively). Assessment included: clinical examination; SARA; 9 holes peg test (9-HPT) for the dominant hand; 6 min walking test (6MWT); Timed 25-Foot Walk (T25-FW); GA. Standardized GA was conducted by an optoelectronic motion capture system with eight-camera (Vicon MX, UK) at the sampling rate of 200 Hz, as previously described [5]. Subjects received 33 markers located on anatomical landmarks as indicated by the Plug-in-Gait protocol in order to reconstruct a full body kinematic and kinetic model. Collected data were normalized according to anthropometric features; considered spatio-temporal parameters are indicated in Table 1. The Clinical Global Impression (CGI) index for severity (CGI-S) was collected at T0; the CGI for improvement (CGI-I) index together with adverse events report were collected at T1-T2 visits. The study followed ethical principles of Helsinki declaration and local ethical standards. Informed consent was signed by participants or their legal representatives.

**Table 1:** n = patient number; M = male, F = female, ST/LT = short/long treatment

n	group	sex	variants				age		onset	phenotype				
1	ST	M	c.901C > T; p.R301W c.1331_1332insCACAG; p.Glu446AlafsTer33				10		3	ataxia, tremor, epilepsy, mild intelectual retardation				
2	ST	M	c.901C > T; p.R301W c.1331_1332insCACAG p.Glu446AlafsTer33				7		3	ataxia, mild intelectual retardation				
3	LT	F	c.1844G > A; p.G615D - c.589-3C > G p.Leu197Valfs*20				8		6	ataxia, tremor				
4	LT	F	c.901C > T; p.R301W c.589-3C > G (splice)				13		2	epilepsy, mild intelectual retardation				
	T0 - Baseline													
n		SARA	6MWT	9-HPT	T25-FW	Foot Off (%gait cycle)	Stride Velocity (m/sec)	Stride Length (m)	Stride Time (sec)	Stride Width (m)	Step Length (m)	Stance Time (sec)	Swing Time (sec)	Double support (sec)
1		11,50	419,55	39,10	6,65	58,67	1,06	1,10	1,03	0,20	0,55	0,61	0,43	0,08
2		14,50	403,28	47,00	7,80	57,58	0,68	0,76	1,14	0,21	0,41	0,65	0,49	0,10
3		10,00	468,40	38,60	6,30	61,48	1,07	1,15	1,07	0,16	0,58	0,66	0,41	0,12
4		10,00	507,60	43,80	5,09	58,28	1,07	1,02	0,96	0,19	0,51	0,56	0,40	0,07
ST	mean	13,00	411,42	43,05	7,23	58,13	0,87	0,93	1,09	0,21	0,48	0,63	0,46	0,09
	st.dev	2,12	11,50	5,59	0,81	0,77	0,27	0,23	0,08	0,00	0,10	0,03	0,04	0,01
LT	mean	10,00	488,00	41,20	5,70	59,88	1,07	1,09	1,02	0,18	0,54	0,61	0,41	0,10
	st.dev	0,00	27,72	3,68	0,86	2,26	0,00	0,09	0,08	0,02	0,05	0,07	0,01	0,04
T1–6 months														
ST	mean	13,75	443,15	40,80	6,38	60,12	0,86	0,94	1,09	0,16	0,46	0,66	0,44	0,11
	st.dev	1,77	8,98	1,27	1,04	1,53	0,15	0,15	0,01	0,01	0,10	0,03	0,01	0,03
LT	mean	9,75	454,75	40,09	5,60	57,24	1,22	1,18	0,98	0,20	0,59	0,56	0,42	0,07
	st.dev	1,06	7,42	0,26	0,76	2,04	0,13	0,02	0,12	0,02	0,01	0,09	0,03	0,01
T2–12 months														
ST	mean	14,25	431,00	41,95	11,5	59,05	1,01	1,00	1,01	0,16	0,50	0,60	0,41	0,11
	st.dev	1,06	35,36	1,48	0,71	2,66	0,14	0,01	0,16	0,03	0,02	0,12	0,04	0,04
LT	mean	9,00	475,25	41,14	5,39	59,03	1,19	1,14	0,96	0,18	0,57	0,57	0,39	0,10
	st.dev	0,00	29,91	0,33	0,54	3,02	0,01	0,07	0,07	0,03	0,04	0,07	0,00	0,04

### Statistical analysis

Data distribution was assessed by the Shapiro-Wilk test. Variable were compared by one-way-ANOVA or non-parametric test, as appropriate. Short-term clinical efficacy (6 months) was measured by one-way-ANOVA between T0 and T1 clinical/GA values. The long-term efficacy (1 year) was assessed by mixed-ANOVA, with TIME (T0, T1, T2) as independent variable, and GROUP as within-subject factor. Mauchley’s test examined for sphericity, using the Greenhouse-Geisser correction for non-spherical data.

Statistical significance was set at *p* < 0.05. Analysis was conducted by IBM-SPSS software.

## Results

Table 1 summarizes clinical and genetic features of patients. They all presented similar phenotypes with mild ataxia, absent/minimal intellectual delay or tremor (not interfering with evaluations), no neuropathy, no brain lesions (apart subtle cerebellar atrophy). Only one patient had epilepsy, controlled by levetiracetam.

**Fig. 1:**
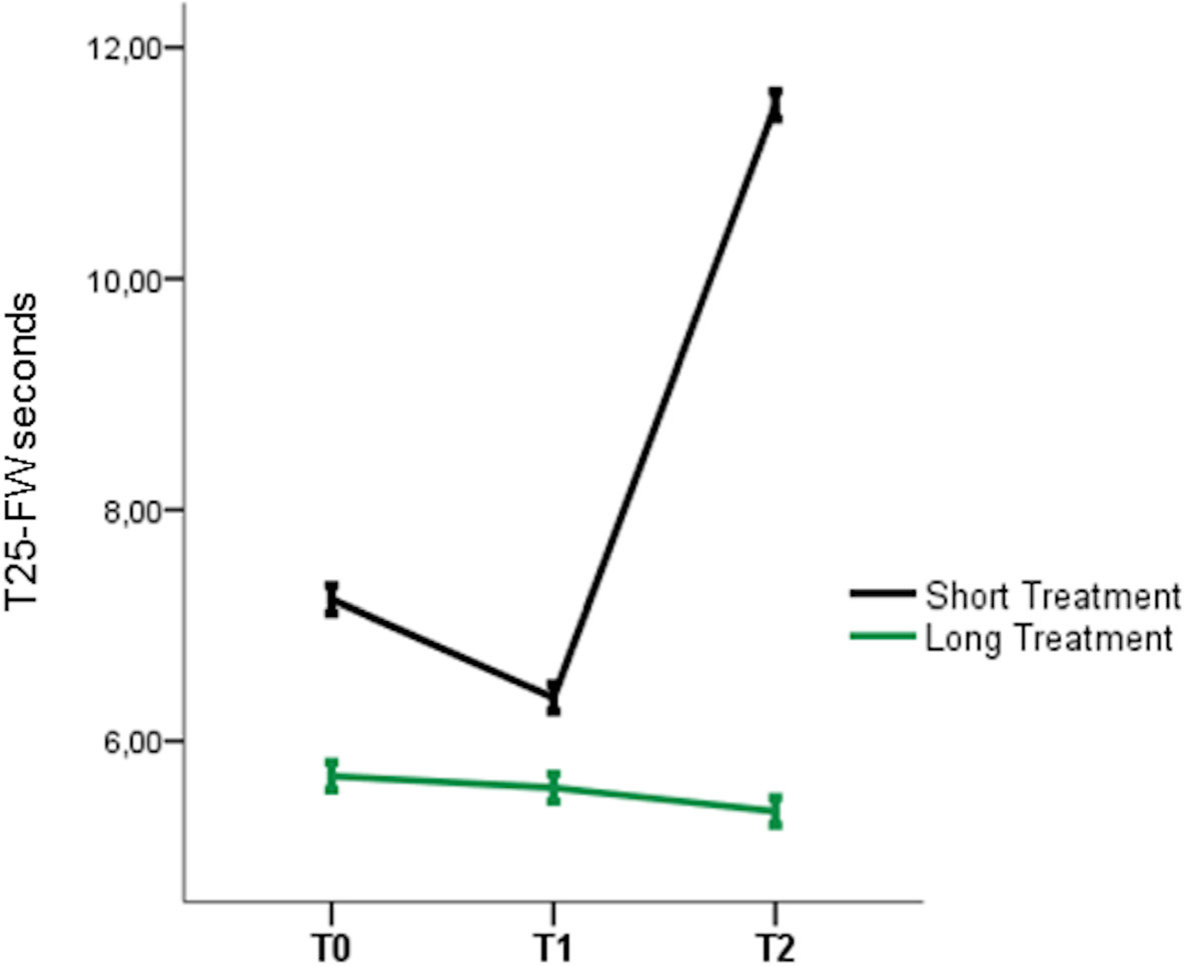
One year T25-FW significant changes

All patients maintained CoQ10 supplementation up to T1. Patients 3–4 (**“**long treatment”, LT) continued up to T2, in the absence of adverse events. Patients 1–2 discontinued in the 7th month (“short treatment”, ST), because of gastrointestinal disturbances and poor subjective improvement. Such a discontinuation allowed evaluating the one-year motor outcome based on duration of CoQ10 supplementation. In fact, mixed ANOVA revealed significant TIME and TIMEXGROUP effects on the T25-FW, (F [2, 4] = 13.59, *p* = 0.016, ƞ^2^ = 0.87) and (F [2, 4] = 8.325, *p* = 0.012, ƞ^2^ = 0.867) respectively **(**Fig. 1**)**, with significance resulting at T1-T2 interval (*p* = 0.01). Specifically, the T25-FW test time increased in ST group and decreased in LT group. Also SARA score tended to decrease in LT group (10% reduction), although without statistical significance. Conversely, the 6-months long treatment did not change clinical and GA parameters in the whole population. No differences resulted at baseline between LT and ST (Table 1 reports all clinical/GA values). At GCI scale, patient 3–4 showed both “minimally improvement” a T2 evaluation; patient 1 was “no change”; patient 2 was “minimally worse”.

## Discussion

This study described the long-term follow up of a selected and homogeneous pediatric population of ARCA2 patients receiving CoQ10 standard oral supplementation (15 mg/kg/day). The main outcome was the motor performance, since we systematically assessed ataxia, coordination and gait with both clinical scores and videoelectronic GA, which in turn provides a more objective and reliable measurement of kinematic parameters [5].

The cohort was observed for one year; however, two patients (ST group) discontinued earlier the treatment (7th month). This allowed us to compare the outcome between ST and LT patients, highlighting the importance of treatment duration in the response to CoQ10 supplementation. In facts, patients taking the one-year-long supplementation (LT group) and those interrupting (ST group) significantly differed in the T25-FW test, showing better performance the first and worst the latter. As well, the LT group ameliorated in SARA score (although not significantly) and GCI index.

Such changes in clinical scores were not paralleled by modifications in GA parameters and 6MWT, probably because CoQ10 supplementation induces clinical effects not strictly related to cerebellar ataxia, fluidity of movements and physical endurance. In facts, T25-FW test specifically assesses the gait sprint and often correlates with cognitive processing speed [6, 7]. Conversely, GA and 6MWT typically measure self-selected pace velocity, which represents a different aspect of motor behavior [8].

Primary CoQ10 deficiency due to *ADCK3* variants leads to the degeneration of Purkinje cells and cerebellar atrophy, which mainly accounts for ataxia and other neurological signs [9]. However, other cellular defects, including oxidative stress, mitochondrial and lysosomal impairment, affect ARCA2 patients at systemic level [10]. Therefore, we could suppose that prolonged CoQ10 supplementation operates as a systemic antioxidant or bioenergetics support [11], even at peripheral level, rather than a targeted intervention for cerebellar dysfunction.

These findings are definitely preliminary, since resulting from an open observation on few patients rather than a placebo-controlled trial. Actually, the rarity of the condition limited the sample size. Moreover, the absence of a well-defined natural history of ARCA2 progression prevents solid conclusions.

However, differently form other previous reports, we performed a systematic, comprehensive, objective, long-term evaluation of clinical efficacy of a standard dose of CoQ10, which provides sufficient reliability to our results.

## Conclusions

We found that the CoQ10 supplementation may produce mild clinical benefit in a duration-dependent manner in ARCA2 patients, enhancing general features of motor activity, such as those related to velocity. However, overall efficacy needs to be validated into dedicated clinical trials. While our study suggests that long duration is crucial to reach significant effects, other issues remain to be properly addressed. Specifically, different doses of treatment should be tested and a possible critical time window of intervention identified. Moreover, since ataxia clinical scores and GA parameters resulted uninformative in our experience, other long-term outcomes (e.g. neuropsychological functions) or biomarkers (e.g. CoQ10 peripheral levels [4]) should be considered.

## Data Availability

Data and materials are available from the corresponding author on reasonable request.

## Abbreviations

6MWT: 6 min walking test
9-HPT: 9 holes peg test
ARCA2: Autosomal recessive cerebellar ataxia type 2
CoQ10: Coenzyme Q10
GA: Gait analysis
LT: Long treatment
SARA: Scale for Assessment and Rating of Ataxia
ST: Short treatment
T25-FW: Timed 25-Foot Walk
